# Ascorbic acid in Charcot–Marie–Tooth disease type 1A (CMT-TRI*AA*L and CMT-TRAUK): a double-blind randomised trial

**DOI:** 10.1016/S1474-4422(11)70025-4

**Published:** 2011-04

**Authors:** Davide Pareyson, Mary M Reilly, Angelo Schenone, Gian Maria Fabrizi, Tiziana Cavallaro, Lucio Santoro, Giuseppe Vita, Aldo Quattrone, Luca Padua, Franco Gemignani, Francesco Visioli, Matilde Laurà, Davide Radice, Daniela Calabrese, Richard AC Hughes, Alessandra Solari, for the CMT-TRIAAL and CMT-TRAUK groups

**Affiliations:** a Istituto di Ricovero e Cura a Carattere Scientifico (IRCCS) Foundation, Carlo Besta Neurological Institute, Milan, Italy; b Medical Research Council Centre for Neuromuscular Diseases, University College London Institute of Neurology, London, UK; c Department of Neurology, Ophthalmology, and Genetics, University of Genoa, Genoa, Italy; d Department of Neurological, Neuropsychological, Morphological, and Motor Sciences, University of Verona, Verona, Italy; e Department of Neurological Sciences, Federico II University of Naples, Naples, Italy; f Department of Neurosciences, Psychiatry, and Anaesthesiology, University of Messina, Messina, Italy; g Department of Medical Sciences, Institute of Neurology, University Magna Graecia, Catanzaro, Italy; h Neuroimaging Research Unit, National Research Council, Catanzaro, Italy; i Department of Neuroscience, Institute of Neurology, Catholic University, Rome, Italy; j Don Gnocchi Foundation, Milan, Italy; k Department of Neurosciences, University of Parma, Parma, Italy; l Department of Pharmacological Sciences, University of Milan, Milan, Italy; m Madrid Institute for Advanced Studies on Food (IMDEA Food), Madrid, Spain; n Department of Epidemiology and Biostatistics, European Institute of Oncology, Milan, Italy

## Abstract

**Background:**

Ascorbic acid reduced the severity of neuropathy in transgenic mice overexpressing peripheral myelin protein 22 (*PMP22*), a model of Charcot–Marie–Tooth disease type 1A (CMT1A) associated with the *PMP22* duplication. However, in three 1-year trials, ascorbic acid had no benefit in human beings. We did a multicentre 2-year trial to test the efficacy and tolerability of ascorbic acid in patients with CMT1A.

**Methods:**

Adult patients (aged 18–70 years) with symptomatic CMT1A were enrolled from nine centres in Italy and the UK, and were randomly assigned (1:1 ratio) to receive 1·5 g/day oral ascorbic acid or matching placebo for 24 months. The randomisation sequence was computer generated by block randomisation, stratified by centre and disease severity, and patients were allocated to treatment by telephone. The primary outcome was change in the CMT neuropathy score (CMTNS) at 24 months. Secondary outcomes were timed 10 m walk test, nine-hole peg test, overall neuropathy limitations scale, distal maximal voluntary isometric contraction, visual analogue scales for pain and fatigue, 36-item short-form questionnaire, and electrophysiological measurements. Patients, treating physicians, and physicians assessing outcome measures were masked to treatment allocation. Analysis of the primary outcome was done on all randomised patients who received at least one dose of study drug. This study is registered, numbers ISRCTN61074476 (CMT-TRAUK) and EudraCT 2006-000032-27 (CMT-TRI*AA*L).

**Findings:**

We enrolled and randomly assigned 277 patients, of whom six (four assigned to receive ascorbic acid) withdrew consent before receiving treatment; 138 receiving ascorbic acid and 133 receiving placebo were eligible for analysis. Treatment was well tolerated: 241 of 271 patients (89% in each group) completed the study; 20 patients (nine receiving ascorbic acid) dropped out because of adverse events. Mean CMTNS at baseline with missing data imputed was 14·7 (SD 4·8) in the ascorbic acid group and 13·9 (4·2) in the placebo group. Mean worsening of CMTNS was 0·2 (SD 2·8, 95% CI −0·3 to 0·7) in the ascorbic acid group and 0·2 (2·7, −0·2 to 0·7) in the placebo group (mean difference 0·0, 95% CI −0·6 to 0·7; p=0·93). We recorded no differences between the groups for the secondary outcomes at 24 months. 21 serious adverse events occurred in 20 patients, eight in the ascorbic acid group and 13 in the placebo group.

**Interpretation:**

Ascorbic acid supplementation had no significant effect on neuropathy compared with placebo after 2 years, suggesting that no evidence is available to support treatment with ascorbic acid in adults with CMT1A.

**Funding:**

Telethon-UILDM and AIFA (Italian Medicines Agency) for CMT-TRI*AA*L, and Muscular Dystrophy Campaign for CMT-TRAUK.

## Introduction

Charcot–Marie–Tooth disease (CMT), a disease for which no drug treatments are available, is the most common inherited neuromuscular disorder, and is characterised clinically by distal wasting, weakness, and sensory loss. The main subtype is type 1A (CMT1A), representing about half of all CMT cases. CMT1A is associated with an autosomal dominant duplication of the peripheral myelin protein 22 gene (*PMP22*), causing peripheral nerve demyelination and secondary axonal loss.^1^ Ascorbic acid reduced the severity of neuropathy in transgenic mice overexpressing *PMP22*, an animal model of human CMT1A, compared with untreated mice.^2^ Some clinical indicators improved during treatment, suggesting partial reversion of the phenotype. Ascorbic acid promotes myelination in vitro and possibly decreases *PMP22* expression.^2^, ^3^ Evidence of efficacy of ascorbic acid in the animal model prompted initiation of randomised controlled trials to test the efficacy of ascorbic acid in patients with CMT1A.

Three trials assessing 1-year ascorbic acid treatment in patients with CMT1A have been completed. In a Dutch proof-of-concept trial, ascorbic acid had no effect in five patients (aged 14–24 years) treated with 2 g/day compared with six patients on placebo (aged 13–24 years).^4^ Findings of an Australian trial of 42 children treated with an equivalent dose (30 mg/kg per day) failed to show a significant effect on disease course compared with 39 children on placebo.^5^ Similarly, in a French trial of 179 adult patients treated with one of two doses of ascorbic acid (1 or 3 g/day) or placebo, ascorbic acid had no significant benefit.^6^

In the first two trials, motor conduction velocity of the median nerve was the primary outcome measure, whereas the CMT neuropathy score (CMTNS) was the primary endpoint in the French trial. Two trials reported positive results from post-hoc analyses: in the Australian trial, five children treated with ascorbic acid had a large increase in motor conduction velocity in the median nerve;^5^ and in the French trial, the CMT examination score (CMTES, the clinical component [symptoms, signs] of the CMTNS) was significantly better in the 3 g/day group than in the other groups.^6^

As acknowledged by the authors themselves, these trials were both underpowered and too short.^7^ Moreover, findings obtained from post-hoc analyses should be interpreted with caution.^8^, ^9^ Another crucial point is the choice of motor conduction velocity as the primary endpoint for the Dutch and Australian trials because, although axonal damage correlates with clinical progression, the correlation of motor conduction velocity with clinical progression is controversial.^1^ The only validated CMT-specific outcome measure is the CMTNS, which is a composite of symptoms, signs, and motor and sensory action potential amplitudes of the ulnar or median nerve.^10^ The scale ranges from 0 (normal) to 36 (worst). CMTNS progression rate was 0·68 points per year in a North American study of adults with untreated CMT1A^11^ and 0·5 points per year in the placebo group of the French trial.^6^

We did a phase 3, multicentre, placebo-controlled, double-blind randomised trial to assess the efficacy and tolerability of chronic ascorbic acid treatment in patients with CMT1A in Italy (CMT-TRial Italian with Ascorbic Acid Long term [CMT-TRI*AA*L]) and the UK (CMT-TRial with Ascorbic acid United Kingdom [CMT-TRAUK]). In accordance with the consensus from the 136th European Neuromuscular Centre (ENMC) workshop that the CMTNS was the most appropriate primary endpoint for a trial of ascorbic acid in CMT1A, we chose the CMTNS as our primary outcome.^7^ Our secondary outcomes were also those suggested at this workshop.^7^, ^12^

## Methods

### Patients

Detailed methods are reported in the published protocol.^12^ Patients with clinical and genetic diagnosis of CMT1A were enrolled from eight Italian hospitals and one UK hospital. Eligible patients were aged 18–70 years and had a CMTNS between 1 (excluding the electrophysiological component) and 35 (including the electrophysiological component). Exclusion criteria were contraindications to ascorbic acid (nephrolithiasis, glucose-6-phosphate dehydrogenase deficiency, or iron overload), extra-dietary ascorbic acid supplementation in the 3 months before screening, limb surgery in the 6 months before screening or planned for before final assessment, other causes of neuropathy (appropriate blood tests to exclude other causes of neuropathy were done at screening), or other clinically significant neurological or systemic diseases. At screening, women who were pregnant (patient's statement, no pregnancy test required), breastfeeding, or planning to become pregnant during the study were not included. Women of childbearing age were advised to adopt contraception for the entire duration of the study. Women who became pregnant during the study were advised to stop treatment and notify the treating neurologist immediately. They were followed up regularly for the duration of the study to comply with the intention-to-treat design. The protocol was approved by the institutional review board at every site, and patients gave written informed consent before any study-related procedures.

### Randomisation and masking

Patients were randomly assigned (1:1 ratio) to receive either 1·5 g ascorbic acid per day as three 0·5 g tablets (one at breakfast and two at dinner) or matching placebo, for 24 months. Ascorbic acid and placebo tablets had identical appearance, taste, and smell, were centrally blistered, packed, and labelled before being sent to the pharmacy of each study centre. The randomisation sequence was computer generated (by a pseudo-random number generator) by the randomisation unit (Neuroepidemiology Unit, Carlo Besta Neurological Institute, Milan, Italy), which was independent of the study. The sequence was known only by staff at the randomisation unit and the drug dispenser. Treatment was allocated centrally by telephone, and was stratified by centre and disease severity (CMTNS ≤10 *vs* >10, or CMTES ≤8 *vs* >8 if electrophysiological assessment was not done in the previous 3 months), with a block size of four (unknown to investigators) within each centre. The sequence was available in opaque sealed envelopes at every centre for emergency unmasking. Patients, treating physicians, and physicians assessing outcomes with clinical scales at baseline and during follow-up were masked to treatment allocation.

### Procedures

We decided to give 1·5 g/day ascorbic acid, subdivided into two doses, on the basis of available pharmacokinetic data showing cellular saturation at doses of 100 mg/day,^13^ the US Institute of Medicine recommendations of a tolerable upper intake level of 2 g/day,^14^ and the dose given to mice.^2^, ^12^

After providing informed consent, patients underwent physical examination and laboratory testing at screening. Eligible patients returned at visit 1 (baseline) and were randomly assigned to treatment groups. Study visits were done at 6, 12, 18, and 24 months. Drug compliance, concomitant drug use, clinical scales, and electrophysiology were assessed at baseline and every 6 months. Clinical scales assessed were CMTNS, maximal voluntary isometric contraction with a hand-held myometer (Cit Technics, Haren, Netherlands) for distal arm movements (hand-grip and large three-point pinch) and leg movements (foot dorsiflexion and plantar flexion, by use of a stabilising device),^18^ overall neuropathy limitations scale,^19^ timed 10 m walk test, and nine-hole peg test.^12^ The 36-item short-form questionnaire (SF-36)^15^, ^16^ and pain and fatigue visual analogue scales^12^, ^17^ were used at baseline and at 12 and 24 months.^12^ Safety was assessed by physical examination, adverse events, and serious adverse events every 6 months, and by laboratory tests at baseline and at 6, 12, and 24 months.

At every visit, we took electrophysiological recordings from the motor ulnar, median, and peroneal nerves and the sensory ulnar nerve (non-dominant side, antidromic technique), and differences between baseline and negative peak amplitudes of compound motor action potentials and sensory action potentials were assessed. Foot and hand skin temperature was checked and kept at 32–34°C. Patients had laboratory tests of blood cell count, urine and its sediment, serum iron, total iron binding capacity, ferritin, and uric acid. Adherence was assessed by counting tablet returns every 3 months (including visits or contacts for drug delivery at 3, 9, 15, and 21 months), and measurement of serum ascorbic acid concentrations by spectrophotometry^20^ every 6 months. Patients taking less than 80% of tablets were judged to be non-compliant.

The primary outcome was change in CMTNS at 2 years; for CMTNS, CMTES, and CMTNS subitems, data are presented at 12 and 24 months. Secondary outcomes were timed 10 m walk test, nine-hole peg test, overall neuropathy limitations scale, distal maximal voluntary isometric contraction, visual analogue scales for pain and fatigue, SF-36, and electrophysiological measurements; for these outcomes, data are presented at 24 months. An independent data and safety monitoring committee supervised the safety and data quality, and did a masked interim analysis of the primary outcome measure at 1 year, to confirm or disconfirm study continuation.

### Statistical analysis

On the basis of longitudinal data,^11^ we defined a significant difference as an improvement in CMTNS by 0·5 points or more in the ascorbic acid group compared with a worsening by 1 point in the placebo group at 24 months. Therefore, we expected a CMTNS of 13·0 in the ascorbic acid group and 14·5 in the placebo group at 24 months, with a common SD of 6·5.^10^ With these values, a two-sided α of 0·05, and the assumption that 20% of patients would be lost to follow-up or discontinue treatment, we calculated that a sample of 272 patients (136 per group) would provide 90% statistical power to detect a significant difference between the ascorbic acid and placebo groups.

The main analysis of the primary outcome included all randomised patients who took at least one dose of study treatment. We compared the study groups for the primary outcome by use of a linear mixed model for longitudinal data. To keep imputed values to a minimum, patients who stopped treatment were asked to attend scheduled follow-up visits. For patients who were unavailable, missing data were imputed according to Rubin's multiple imputation approach. A per-protocol analysis was done for the primary outcome and all secondary outcomes, and included patients who accomplished the outcome measures and were compliant with treatment (≥80% of tablets). For analysis of the primary outcome, we used the covariates baseline CMTNS, centre, age, and sex, and we also tested for first-order interaction terms (centre per treatment and sex per treatment). Between-group comparisons of proportions were done with Pearson's χ^2^ test or Fisher's exact test, and comparisons of continuous variables were done with the Wilcoxon rank-sum test. Two-sided p values of less than 0·05 were judged to be significant; p values were not adjusted for multiple comparisons. Analyses were done with Stata (version 10.0) and SAS (version 9.2).

This study is registered, numbers ISRCTN61074476 (CMT-TRAUK) and EudraCT 2006-000032-27 (CMT-TRI*AA*L). CMT-TRI*AA*L and CMT-TRAUK shared a common protocol but have different names and registration numbers because funding was separate in Italy and the UK.

### Role of the funding source

A representative of the main sponsor (Telethon Italy) attended meetings of the steering committee as non-voting external participant. The study database is held by the Neuroepidemiology Unit, Carlo Besta Neurological Institute, Milan, Italy. The funding sources had no role in the study design, data collection, data analysis, data interpretation, preparation of the report, or the decision to submit for publication. The corresponding author and all coauthors had access to all data and had final responsibility for the decision to submit for publication.

## Results

**Figure 1:**
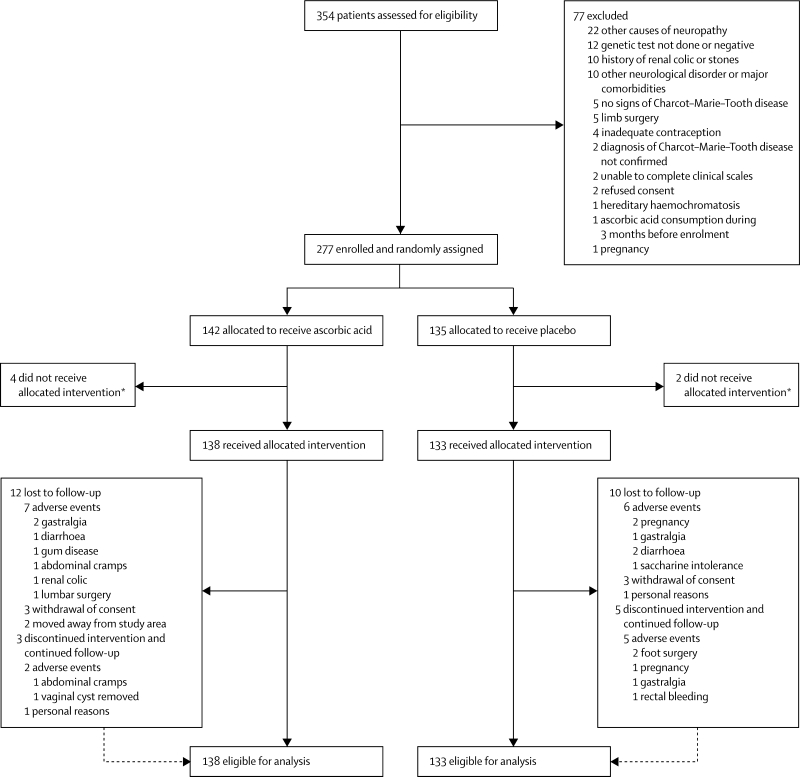
Trial profile *Consent withdrawn before start of treatment.

Between March, 2006, and September, 2007, 277 of 354 patients (78%) were deemed eligible and were randomly assigned, of whom six withdrew consent before receiving treatment; 138 assigned to receive ascorbic acid and 133 assigned to receive placebo were eligible for the main analysis (figure 1). Demographic and clinical characteristics were balanced across treatment groups at baseline (table 1). Only one patient in the ascorbic acid group had a CMTNS of more than 30, indicating severe disease, five patients (three in the ascorbic acid group) had a CMTNS of more than 25, and two patients (one in each group) had a CMTNS of less than 5.

**Table 1:** Demographic and clinical characteristics at baseline

		**Ascorbic acid**			**Placebo**		
		Patients	Mean (SD)	Median (IQR)	Patients	Mean (SD)	Median (IQR)
Women		78/138 (57%)	..	..	85/133 (64%)	..	..
Age (years)		138	43·8 (13·7)	45·2 (32·1–54·5)	133	41·2 (12·4)	41·8 (31·8–51·3)
CMTNS							
	Total score	137	14·6 (4·9)	14·0 (11·0–18·0)	131	13·9 (4·3)	13·0 (11·0–17·0)
	CMTES	138	9·2 (4·0)	8·5 (6·0–11·0)	133	8·6 (3·6)	8·0 (6·0–11·0)
	Neurophysiological component	137	5·5 (1·6)	6·0 (5·0–7·0)	131	5·2 (1·6)	5·0 (5·0–6·0)
Timed 10 m walk test (s)		138	9·3 (5·1)	7·8 (6·8–9·7)	133	9·0 (5·4)	7·4 (6·5–8·7)
Nine-hole peg test (s; average for both sides)		137	24·8 (7·6)	23·1 (20·1–26·6)	131	23·4 (5·7)	22·1 (19·9–24·6)
Overall neuropathy limitations scale score		138	..	4·0 (2·0–4·0)	133	..	3·0 (2·0–4·0)
Maximal voluntary isometric contraction (N)							
	Handgrip	138	89·0 (45·6)	86·5 (51·0–115·0)	132	85·8 (38·8)	77·0 (58·5–110·5)
	Large three-point pinch	138	67·4 (33·2)	64·0 (42·0–89·0)	132	65·2 (29·4)	63·0 (44·5–78·5)
	Foot dorsiflexion	137	67·9 (61·7)	60·0 (23·0–92·0)	131	62·8 (43·1)	56·0 (28·0–87·0)
	Foot plantar flexion	138	104·3 (69·3)	90·0 (59·0–130·0)	131	97·0 (59·7)	84·0 (59·0–128·0)
Visual analogue scale score							
	Pain	137	3·7 (3·1)	4·0 (0·5–6·0)	133	3·6 (2·9)	3·0 (1·0–6·0)
	Fatigue	137	5·0 (2·7)	5·0 (3·0–7·0)	133	4·8 (2·9)	5·0 (2·5–7·0)
SF-36 score							
	Physical functioning	133	61·3 (25·7)	60·0 (45·0–85·0)	132	62·9 (25·7)	65·0 (42·5–85·0)
	Bodily pain	136	63·5 (28·4)	62·0 (41·0–100)	133	63·6 (25·8)	62·0 (41·0–84·0)
	Energy	134	50·4 (21·3)	50·0 (35·0–65·0)	130	52·4 (21·6)	55·0 (40·0–65·0)
CMAP summatory (mV)		135	6·6 (3·9)	6·5 (3·8–9·3)	128	7·1 (4·1)	7·2 (4·3–9·2)
MCV (m/s)*		124	20·1 (4·8)	20·0 (17·1–23·0)	118	20·6 (4·4)	20·4 (17·6–23·3)

Higher CMTNS, CMTES, or overall neuropathy limitations scale score indicates more severe disease; higher visual analogue scale score indicates more severe symptom (pain or fatigue); higher SF-36 score indicates better quality of life. CMTNS=Charcot–Marie–Tooth neuropathy score. CMTES=Charcot–Marie–Tooth examination score. N=Newtons. SF-36=36-item short-form questionnaire. CMAP summatory=sum of compound motor action potentials of the three motor nerves (ulnar, median, and peroneal nerves). MCV=motor conduction velocity.

* Mean from median and ulnar nerves.

**Figure 2:**
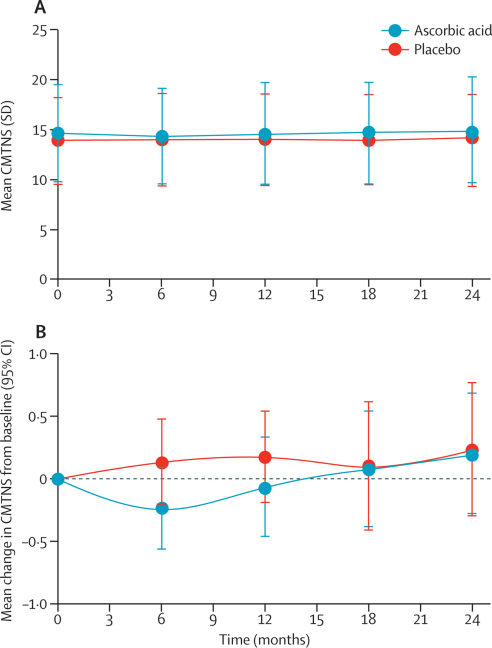
Mean CMTNS (A) and mean change in CMTNS from baseline (B) Analysis was done on all patients who were randomised and received at least one dose of study drug. Higher CMTNS indicates more severe disease. CMTNS=Charcot–Marie–Tooth neuropathy score.

In the main analysis of the primary outcome with missing data imputed, mean CMTNS changed from 14·7 (SD 4·8) at baseline to 14·9 (5·4) at 24 months in the ascorbic acid group, and from 13·9 (4·2) to 14·2 (4·4) in the placebo group (figure 2A). Mean change in CMTNS from baseline to 24 months was 0·2 (SD 2·8, 95% CI −0·3 to 0·7) in the ascorbic acid group and 0·2 (2·7, −0·2 to 0·7) in the placebo group, with a mean between-group difference of 0·0 (95% CI −0·6 to 0·7; p=0·93; figure 2B). Mean change in CMTNS from baseline to 12 months was −0·1 (SD 2·4, 95% CI −0·5 to 0·3) in the ascorbic acid group and 0·2 (2·1, −0·2 to 0·5) in the placebo group, with a mean between-group difference of 0·2 (95% CI −0·3 to 0·8; p=0·39). Data were missing for three patients at baseline (one on ascorbic acid *vs* two on placebo), 25 patients at each of 6 months (12 *vs* 13) and 12 months (13 *vs* 12), 34 patients at 18 months (16 *vs* 18), and 26 patients at 24 months (12 *vs* 14). Findings of the per-protocol analysis of the primary outcome of CMTNS showed a worsening of disease in both the ascorbic acid and placebo groups at 24 months (table 2). CMTNS was higher in the placebo group than in the ascorbic acid group at both 12 and 24 months, with a mean between-group difference of 0·3 (95% CI −0·3 to 0·9; p=0·27) at 12 months and 0·4 (−0·3 to 1·1; p=0·28) at 24 months.

**Table 2:** Change in CMTNS, CMTES, and CMTNS subitems from baseline to 12 and 24 months (per-protocol population)

	**Ascorbic acid**		**Placebo**		**p value**
	Patients	Mean change (SD, 95% CI)	Patients	Mean change (SD, 95% CI)	
**CMTNS, total score**					
12 months	122	−0·1 (−0·5 to 0·3)	116	0·2 (−0·2 to 0·6)	0·27
24 months	118	0·1 (−0·4 to 0·6)	109	0·5 (0·0 to 1·0)	0·28
**CMTES**					
12 months	122	0·2 (−0·2 to 0·5)	116	0·2 (−0·1 to 0·6)	0·52
24 months	118	0·2 (−0·3 to 0·6)	109	0·5 (0·1 to 0·9)	0·17
**CMTNS, symptoms**					
12 months	122	0·0 (−0·2 to 0·3)	116	0·1 (−0·1 to 0·3)	0·68
24 months	118	0·1 (−0·2 to 0·3)	109	0·1 (−0·2 to 0·3)	0·87
**CMTNS, signs**					
12 months	122	0·1 (−0·2 to 0·4)	116	0·2 (−0·1 to 0·5)	0·44
24 months	118	0·1 (−0·2 to 0·4)	109	0·5 (0·2 to 0·7)	0·11
**CMTNS, neurophysiological component**					
12 months	122	−0·3 (−0·5 to −0·1)	116	0·0 (−0·3 to 0·2)	0·06
24 months	118	−0·1 (−0·3 to 0·1)	109	−0·1 (−0·3 to 0·2)	0·84

Higher CMTNS or CMTES indicates more severe disease. CMTNS=Charcot–Marie–Tooth neuropathy score. CMTES=Charcot–Marie–Tooth examination score.

In the prespecified multivariate analysis, ascorbic acid treatment had no significant effect on the primary outcome (p=0·491), and had no interaction with sex (p=0·891) or centre (p=0·529; webappendix p 1). Greater CMTNS at baseline (p<0·0001), older age (p=0·005), and longer time from start of study (p=0·004) were associated with worse prognosis. Centre had a significant effect (p=0·042): in a subgroup analysis, patients treated at the centres in Milan and the UK had a slightly better outcome, irrespective of treatment group, than did those treated at the remaining seven centres, but this difference was significant only at 12 months (p=0·04). This marginal difference, which was not significant at 24 months, does not affect interpretation of treatment effect (webappendix p 1).

Findings of the per-protocol analysis of secondary outcomes showed no significant difference between treatment groups for change in timed 10 m walk test, nine-hole peg test, overall neuropathy limitations scale, maximal voluntary isometric contraction, visual analogue scale scores for pain and fatigue, SF-36 scores, or electrophysiological measurements (table 3). Furthermore, changes from baseline to 24 months were negligible in both groups. Foot dorsiflexion seemed to be the most responsive measure with a mean worsening of more than 10% (Table 1, Table 3).

**Table 3:** Change in secondary outcomes from baseline to 24 months (per-protocol population)

		**Ascorbic acid**		**Placebo**		**p value**
		Patients	Mean change (95% CI)	Patients	Mean change (95% CI)	
Timed 10 m walk test (s)		118	0·76 (0·08 to 1·44)	109	1·12 (−0·38 to 2·61)	0·81
Nine-hole peg test (s; average of both sides)		118	0·11 (−0·59 to 0·80)	106	0·85 (0·33 to 1·37)	0·07
Overall neuropathy limitations scale score*		118	0·11 (−0·07 to 0·29)	109	0·09 (−0·07 to 0·25)	0·99
Maximal voluntary isometric contraction (N)						
	Handgrip	118	−6·23 (−10·37 to −2·09)	107	−6·86 (−10·70 to −3·02)	0·83
	Large three-point pinch	118	−2·00 (−4·83 to 0·83)	107	−3·62 (−7·19 to −0·05)	0·97
	Foot dorsiflexion	118	−9·19 (−16·26 to −2·12)	107	−9·81 (−14·30 to −5·33)	0·25
	Foot plantar flexion	118	−5·78 (−16·34 to 4·78)	107	−2·69 (−11·74 to 6·35)	0·89
Visual analogue scale score						
	Pain	118	0·31 (−0·15 to 0·76)	107	0·59 (0·05 to 1·13)	0·32
	Fatigue	118	−0·33 (−0·82 to 0·17)	107	0·03 (−0·52 to 0·59)	0·41
SF-36 score						
	Physical functioning	115	−0·43 (−3·38 to 2·52)	106	−1·05 (−3·97 to 1·88)	0·81
	Bodily pain	117	−1·27 (−5·42 to 2·87)	107	−0·85 (−5·62 to 3·93)	0·74
	Energy	112	1·21 (−1·97 to 4·38)	104	−0·34 (−3·89 to 3·21)	0·31
CMAP summatory (mV)		113	0·47 (0·01 to 0·92)	104	0·24 (−0·31 to 0·79)	0·43
MCV (m/s)†		107	0·38 (−0·06 to 0·82)	98	0·61 (0·15 to 1·06)	0·88

Higher overall neuropathy limitations scale score indicates more severe disease; higher visual analogue scale score indicates more severe symptom (pain or fatigue); higher SF-36 score indicates better quality of life. N=Newtons. SF-36=36-item short-form questionnaire. CMAP summatory=sum of compound motor action potentials of the three motor nerves (ulnar, median, and peroneal nerves). MCV=motor conduction velocity.

* Data are median change (95% CI).

† Mean from median and ulnar nerves.

Overall, ascorbic acid treatment was well tolerated. 15 patients (11%) in each treatment group discontinued treatment early, of whom six in the ascorbic acid group and five in the placebo group discontinued because of oral or gastrointestinal symptoms (figure 1). 134 patients (97%) on ascorbic acid and 127 (95%) on placebo had drug adherence of more than 80%. 21 serious adverse events occurred in 20 patients, eight events in seven patients on ascorbic acid and 13 events in 13 patients on placebo: three pregnancies; 12 hospital admissions for surgery (four for foot surgery, three for haemorrhoids, and one each for disc herniation, gallbladder stones, hysterectomy, vaginal cyst removal, and tooth extraction); one diagnostic procedure (arteriography); and five hospital admissions for other reasons (depression, loss of consciousness, bronchitis, anaemia, and rectal bleeding). All serious adverse events were deemed to be unrelated to treatment, with the exception of one hospital admission owing to anaemia in a woman on placebo. In eight patients, two receiving ascorbic acid and six receiving placebo, a serious adverse event was the reason for discontinuation of intervention, of whom three patients (one receiving ascorbic acid and two receiving placebo) withdrew from the study. The most common solicited adverse events were gastrointestinal disturbances, which occurred with similar frequency in both groups (webappendix pp 2–7).

Mean serum concentrations of ascorbic acid at baseline were similar in the two study groups: 38·8 μmol/L (SD 29·3, n=88 patients) in the ascorbic acid group, and 34·3 μmol/L (19·8, n=87) in the placebo group (p=0·390). Mean serum concentrations of ascorbic acid in patients receiving ascorbic acid were significantly increased at 24 months (49·1 μmol/L [SD 23·0], n=56) compared with at baseline (p=0·024), and compared with patients receiving placebo at 24 months (37·0 μmol/L [SD 16·2], n=56; p=0·004).

## Discussion

In this 2-year randomised trial of high-dose ascorbic acid in the largest cohort of adults with CMT1A so far, we identified no evidence that ascorbic acid is efficacious for the primary outcome, CMTNS, or for several prespecified secondary outcomes, which included impairment, activity limitation, and quality-of-life measures (panel).

Panel **Research in context**

**Systematic review**

We searched the Cochrane Central Register of Controlled Trials (CENTRAL) on the Cochrane Library (issue 1, 2011), Medline (1985–2011), Embase (1985–2011), and the trial registries ClinicalTrials.gov and Current Controlled Trials with the search terms “peripheral neuropathy”, “hereditary neuropathy”, “Charcot–Marie–Tooth”, “HMSN”, “ascorbic acid”, and “vitamin C”. We also obtained information from handsearching of references in selected articles and conference proceedings, and from contacts with other investigators. We identified seven studies assessing the efficacy of ascorbic acid in patients with CMT1A.^21^ Three are published randomised controlled trials assessing 1-year treatment with ascorbic acid treatment versus placebo, and ascorbic acid did not have a treatment effect in any of the trials.^4^, ^5^, ^6^

**Interpretation**

We identified no evidence of efficacy of 1·5 g/day ascorbic acid in a large cohort of patients with CMT1A across a treatment period of 2 years. We feel that this study adds strong weight to existing evidence that ascorbic acid is not efficacious in CMT1A.

Our findings confirm and expand the results of the Dutch,^4^ Australian,^5^ and French^6^ 1-year studies of ascorbic acid in CMT1A. These studies were limited by their statistical power and, in two studies, by the use of motor conduction velocity as the primary outcome.^4^, ^5^ We addressed these issues by choosing the CMTNS as our primary outcome,^7^ and by powering our trial in accordance with the predicted change in CMTNS from Shy and colleagues' study^11^ (1·4 points in 24 months). The most likely interpretation of our results is that ascorbic acid is not efficacious in CMT1A. Three less likely interpretations are that the study was not long enough, the dose of ascorbic acid was insufficient, or the effect was too small to be detected by the outcome measures used.

With respect to study length, an unexpected finding was that disease progression was slower than was predicted from previous retrospective observational studies.^11^, ^22^ Mean CMTNS worsened by 0·46 points in 24 months (ie, 0·23 points per year) in the placebo group (per-protocol analysis), which is three times lower than that reported in the longitudinal study by Shy and colleagues (0·68 points per year),^11^ lower than that in the placebo group of the French trial (0·5 points per year),^6^ and slightly lower than that reported in a longitudinal study by Verhamme and colleagues (1·5 points worsening of an adapted CMTNS in 5 years).^22^ However, the studies by Shy^11^ and Verhamme^22^ and their colleagues were partly retrospective, and therefore might not be directly comparable with our study. We feel that our large series is highly representative of the adult population with CMT1A treated at neurological centres.

The slow progression of CMTNS in adults with CMT1A raises the possibility that we might have missed a small effect of ascorbic acid in a 2-year study. Increasing the trial length in adults would be very difficult and perhaps impractical, but improved outcome measures could help. Participants of an ENMC workshop in 2009 agreed that some CMTNS components are not sensitive, and a modified CMTNS is in development.^21^ Furthermore, in our study, results indicated that development of myometry (especially foot dorsiflexion) as an outcome measure might be useful. The disease starts in childhood, so treatment effects might be more easily detected in children than in adults in future trials. However, existing evidence suggests that use of CMTNS is not appropriate in children younger than 10 years, and a paediatric CMTNS is in development.^21^

With respect to the dose of ascorbic acid, we chose a dose that was about twice or thrice the equivalent dose given to mice (which synthesise ascorbic acid)^2^ and was expected to achieve maximum and steady plasma concentrations with a low risk of side-effects.^12^ This dose was well tolerated in our study. Although we feel that the dose we used should have been sufficient to detect efficacy, Micallef and colleagues^6^ recorded weak evidence of an effect of 3 g/day ascorbic acid in the French trial, only because CMTES was significantly better in the 3 g/day group than in the other group. This post-hoc finding should be interpreted with caution, and, notably, the most substantial contribution to the difference was from the CMTNS sensory component, which is the least reliable component.^6^, ^10^ Moreover, findings of pharmacokinetic studies suggest that doses of higher than 500 mg/day do not substantially increase cellular concentrations because of saturation and tight metabolic control,^13^ and, in the French trial,^6^ dose increase from 1 to 3 g/day produced only a slight increase in plasma concentrations. Finally, the US Institute of Medicine has set a tolerable upper intake level of 2 g/day, above which the risk of gastrointestinal and renal side-effects increases. We judged that our chosen dose was a good compromise by resulting in near saturation of plasma and cellular concentrations without risk of frequent side-effects, which could also unmask treatment. We await with interest the results of the continuing US study of 4 g/day ascorbic acid (NCT00484510)^21^ and also findings of tolerance to treatment because 5 g/day was poorly tolerated in a small study.^23^

With respect to the size of the effect of ascorbic acid, if the effect is so small that only a biomarker can detect it, it would be unlikely to be clinically significant in a short-term study but might be important in the long term. To address this issue, we are studying *PMP22* expression in skin biopsy samples^24^ before and after treatment in a subgroup of patients from our study.

Findings of this study suggest that ascorbic acid is not efficacious in adults with CMT1A. We have shown that a rigorous international randomised trial can be done in CMT. The use of the CMTNS should also provide the opportunity for meta-analysis of the efficacy of ascorbic acid in CMT1A, when data from other trials that use this endpoint become available. Moreover, the data obtained from all these trials will provide unique information about the natural history of CMT1A.

## Web Extra Material

Supplementary webappendix
