# Supplementary material for: Ascorbic acid in Charcot–Marie–Tooth disease type 1A (CMT-TRIAAL and CMT-TRAUK): a double-blind randomised trial
Source: Lancet Neurol. 2011 Apr;10(4):320–8. doi: 10.1016/S1474-4422(11)70025-4 (PMC3154498; doi:10.1016/S1474-4422(11)70025-4)
Supplement: Supplementary webappendix [file mmc1.pdf]

THE LANCET Neurology

Supplementary webappendix

This webappendix formed part of the original submission and has been peer reviewed. We post it as supplied by the authors.

Supplement to: Pareyson D, Reilly MM, Schenone A, et al. Ascorbic acid in Charcot–Marie–Tooth disease type 1A (CMT-TRIAAL and CMT-TRAUK): a double-blind randomised trial. *Lancet Neurol* 2011; published online March 9. DOI:10.1016/S1474-4422(11)70025-4.

Supplementary Table 1

Table S.1 – Results of linear mixed model analysis for longitudinal data (Intention to treat)

Main effects	Degrees of Freedom Numerator - denominator	F-test value	P-Value
CMTNS at baseline	1-257	969.14	< 0.0001
Age	1-257	8.18	0.005
Time	3-807	4.46	0.004
Centre	8-257	2.04	0.042
Gender	1-257	0.78	0.380
Treatment	1-257	0.47	0.491
**2 way Interactions**			
Gender x Treatment	1-257	0.02	0.891
Centre x Treatment	3-807	0.74	0.529

Table S.1b – Results of linear mixed model analysis for longitudinal data (Intention to treat) excluding Milan and UK centres

Main effects	Degrees of Freedom Numerator - denominator	F-test value	P-Value
CMTNS at baseline	1-164	449.90	< 0.0001
Age	1-164	3.53	0.062
Time	3-522	2.96	0.032
Centre	6-164	1.35	0.237
Gender	1-164	0.91	0.341
Treatment	1-164	0.02	0.893
**2 way Interactions**			
Gender x Treatment	1-164	0.01	0.934
Centre x Treatment	3-522	2.19	0.089

Table S.1c – CMTNS at Baseline and changes at 1 and 2 years (Milan and UK vs other centres)

Time	Centre	No.; Mean (95% CI)	P-Value
Baseline	Milan and UK	95; 14.0 (5.2; 13.0 to 15.1)	0.14
	Other centres	176; 14.4 (4.2; 13.8 to 15.1)	
12 months	Milan and UK	95; -0.3 (1.8; -0.6 to 0.1)	0.04
	Other centres	176; 0.2 (2.5; -0.1 to 0.6)	
24 months	Milan and UK	95; -0.2 (2.6; -0.7 to 0.3)	0.08
	Other centres	176; 0.4 (2.7; 0.0 to 0.8)	

Supplementary Table 2 – Solicited* and unsolicited adverse events**

S TABLE 2.1 – Solicited adverse events by time by study arm

	Time visit (months)	Placebo N (%)	Ascorbic acid N (%)	P-value [(a)]
Headache	6	25 (19.4)	25 (19.4)	1.00
	12	25 (19.8)	20 (15.4)	0.41
	18	17 (14.4)	24 (18.9)	0.39
	24	20 (16.1)	19 (15.1)	0.86
Nausea	6	16 (12.4)	13 (9.6)	0.56
	12	10 (7.9)	10 (7.7)	1.00
	18	6 (5.1)	8 (6.3)	0.79
	24	12 (9.7)	12 (9.5)	1.00
Vomiting	6	9 (7.0)	14 (10.3)	0.39
	12	8 (6.4)	9 (6.9)	1.00
	18	4 (3.4)	6 (4.7)	0.75
	24	4 (3.2)	8 (6.4)	0.38
Diarrhoea	6	16 (12.4)	23 (16.9)	0.39
	12	12 (9.5)	16 (12.3)	0.55
	18	7 (5.9)	11 (8.7)	0.47
	24	11 (8.9)	13 (10.3)	0.83
Gastralgia	6	19 (14.7)	20 (14.7)	1.00
	12	15 (11.9)	21 (16.2)	0.37
	18	15 (12.7)	16 (12.6)	1.00
	24	15 (12.1)	12 (9.5)	0.55
Abdominal pain	6	8 (6.2)	11 (8.1)	0.64
	12	11 (8.7)	9 (6.9)	0.65
	18	4 (3.4)	4 (4.7)	0.75
	24	5 (4.0)	8 (6.4)	0.57

(a) Two-sided Fisher's exact test

S TABLE 2.2 – Unsolicited adverse events by time by study arm

ICD-9 Classification	Time visit (months)	Placebo N (%)	Ascorbic acid N (%)	P-value [(a)]
Infectious and parasitic diseases	6	8 (6.2)	11 (8.5)	0.64
	12	6 (4.8)	5 (3.8)	0.77
	18	4 (3.4)	6 (4.7)	0.75
	24	6 (4.8)	6 (4.8)	1.00
Neoplasms	6	1 (0.8)	7 (5.4)	0.07
	12	1 (0.8)	4 (3.1)	0.37
	18	3 (2.5)	4 (3.1)	1.00
	24	1 (0.8)	3 (2.4)	0.62
Endocrine, nutritional, metabolic disease and immunity disorders	6	0	0	-
	12	1 (0.8)	1 (0.8)	1.00
	18	0	2 (1.6)	0.50
	24	0	1 (0.8)	1.00
Diseases of the blood and blood-forming organs	6	2 (1.6)	0	0.49
	12	1 (0.8)	0	0.49
	18	0	1 (0.8)	1.00
	24	0	1 (0.8)	1.00
Mental disorders	6	3 (2.3)	1 (0.8)	0.62
	12	5 (4.0)	2 (1.5)	0.28
	18	3 (2.5)	3 (2.3)	1.00
	24	5 (4.0)	2 (1.6)	0.28
Diseases of the nervous system	6	1 (0.8)	2 (1.6)	1.00
	12	2 (1.6)	1 (0.8)	0.62
	18	1 (0.8)	1 (0.8)	1.00
	24	1 (0.8)	1 (0.8)	1.00
Diseases of the sense organs	6	6 (4.7)	6 (4.7)	1.00
	12	2 (1.6)	3 (2.3)	1.00
	18	5 (4.2)	3 (2.3)	0.49
	24	4 (3.2)	7 (5.6)	0.54
Diseases of the circulatory system	6	5 (3.9)	6 (4.7)	1.00
	12	3 (2.4)	2 (1.5)	0.68
	18	4 (3.4)	0	0.05
	24	4 (3.2)	3 (2.4)	1.00
Diseases of the respiratory system	6	53 (41.1)	43 (33.3)	0.25
	12	26 (20.6)	25 (19.2)	0.88
	18	30 (25.4)	26 (20.3)	0.36
	24	27 (21.8)	36 (28.6)	0.24
Diseases of the digestive system	6	18 (14.0)	23 (17.8)	0.50
	12	9 (7.1)	8 (6.2)	0.81
	18	13 (11.0)	13 (10.2)	0.84
	24	25 (20.2)	10 (7.9)	0.001

(a) Two-sided Fisher's exact test

S TABLE 2.2 – Unsolicited adverse events by time by study arm (continued)

ICD-9 Classification	Time visit (months)	Placebo N (%)	Ascorbic acid N (%)	P-value [(a)]
Diseases of the genitourinary system	6	16 (12.4)	12 (9.3)	0.55
	12	8 (6.3)	4 (3.1)	0.25
	18	4 (3.4)	4 (3.1)	1.00
	24	11 (8.9)	8 (6.3)	0.48
Diseases of the skin and subcutaneous tissue	6	4 (3.1)	11 (8.5)	0.11
	12	3 (2.4)	5 (3.8)	0.72
	18	2 (1.7)	1 (0.8)	0.61
	24	4 (3.2)	2 (1.6)	0.45
Diseases of the musculoskeletal system and connective tissue	6	27 (20.9)	30 (23.3)	0.76
	12	20 (15.9)	15 (11.5)	0.36
	18	16 (13.6)	20 (15.6)	0.72
	24	24 (19.4)	22 (17.5)	0.75
Symptoms, signs and ill-defined conditions	6	38 (29.5)	39 (30.2)	1.00
	12	11 (8.7)	17 (13.1)	0.32
	18	9 (7.6)	19 (14.8)	1.00
	24	12 (9.7)	13 (10.3)	1.00
Injury and poisoning	6	10 (7.8)	3 (2.3)	0.08
	12	6 (4.8)	3 (2.3)	0.33
	18	5 (4.2)	8 (6.3)	0.58
	24	6 (4.8)	10 (7.9)	0.44
Surgical procedures	6	10 (7.8)	7 (13.2)	0.22
	12	4 (3.2)	8 (6.1)	0.38
	18	12 (10.2)	10 (7.8)	0.66
	24	6 (4.8)	10 (7.9)	0.44
Other (b)	6	2 (1.6)	1 (0.8)	1.00
	12	2 (1.6)	0	0.24
	18	1 (0.8)	1 (0.8)	1.00
	24	2 (1.6)	0	0.25

(a) Two-sided Fisher's exact test
(b) Congenital anomalies and conditions originating in the perinatal period

S TABLE 2.3 – Solicited adverse events severity by study arm

	Time Visit (months)	Severity Grade	Placebo N	Ascorbic acid N	P-value [a]
Headache	6	Mild	12	10	
		Moderate	10	14	0.65
		Severe	3	2	
		Overall	25	26	
	12	Mild	15	10	
		Moderate	10	9	0.64
		Severe	0	1	
		Overall	25	20	
	18	Mild	10	18	
		Moderate	6	5	0.48
		Severe	1	1	
		Overall	17	24	
	24	Mild	17	10	
		Moderate	2	9	0.02
		Severe	1	0	
		Overall	20	19	
Nausea	6	Mild	10	9	
		Moderate	3	3	1.00
		Severe	2	1	
		Overall	15	13	
	12	Mild	5	9	
		Moderate	4	1	0.14
		Severe	1	0	
		Overall	10	10	
	18	Mild	4	6	
		Moderate	2	2	1.00
		Severe	0	0	
		Overall	6	8	
	24	Mild	7	7	
		Moderate	3	4	1.00
		Severe	2	1	
		Overall	12	12	

(a) Two-sided Fisher's exact test

S TABLE 2.3 – Solicited adverse events severity by study arm (continued)

	Time Visit (months)	Severity Grade	Placebo N	Ascorbic acid N	P-value [a]
Vomiting	6	Mild	3	8	
		Moderate	4	4	0.53
		Severe	2	2	
		Overall	9	14	
	12	Mild	4	5	
		Moderate	4	2	0.43
		Severe	0	2	
		Overall	8	9	
	18	Mild	4	4	
		Moderate	0	2	0.47
		Severe	0	0	
		Overall	4	6	
	24	Mild	1	5	
		Moderate	1	2	1.00
		Severe	2	1	
		Overall	4	8	
Diarrhoea	6	Mild	10	15	
		Moderate	4	7	0.76
		Severe	2	1	
		Overall	16	23	
	12	Mild	3	8	
		Moderate	7	4	0.23
		Severe	2	4	
		Overall	12	16	
	18	Mild	6	5	
		Moderate	1	6	0.15
		Severe	0	0	
		Overall	4	6	
	24	Mild	6	6	
		Moderate	2	6	0.25
		Severe	3	1	
		Overall	11	13	

(a) Two-sided Fisher's exact test

S TABLE 2.3 – Solicited adverse events severity by study arm (continued)

	Time Visit (months)	Severity Grade	Placebo N	Ascorbic acid N	P-value [(a)]
Gastralgia	6	Mild	14	13	
		Moderate	3	5	0.77
		Severe	1	2	
		Overall	18	20	
	12	Mild	9	14	
		Moderate	6	6	0.84
		Severe	0	1	
		Overall	15	21	
	18	Mild	8	6	
		Moderate	7	9	0.59
		Severe	0	1	
		Overall	15	16	
	24	Mild	6	5	
		Moderate	8	6	1.00
		Severe	1	1	
		Overall	15	12	
Abdominal Pain	6	Mild	4	9	
		Moderate	2	2	0.17
		Severe	2	0	
		Overall	8	11	
	12	Mild	6	6	
		Moderate	4	2	0.81
		Severe	1	1	
		Overall	12	16	
	18	Mild	1	5	
		Moderate	2	1	0.19
		Severe	1	0	
		Overall	4	6	
	24	Mild	2	3	
		Moderate	2	4	1.00
		Severe	1	1	
		Overall	5	8	

(a) Two-sided Fisher’s exact test

*solicited adverse events = adverse events most frequently described associated with AA treatment and explicitly asked for by physician at each patient visit

** unsolicited adverse events = all other adverse events reported by patients
